# Patient-Specific Detection of Cerebral Blood Flow Alterations as Assessed by Arterial Spin Labeling in Drug-Resistant Epileptic Patients

**DOI:** 10.1371/journal.pone.0123975

**Published:** 2015-05-06

**Authors:** Ilaria Boscolo Galazzo, Silvia Francesca Storti, Alessandra Del Felice, Francesca Benedetta Pizzini, Chiara Arcaro, Emanuela Formaggio, Roberto Mai, Michael Chappell, Alberto Beltramello, Paolo Manganotti

**Affiliations:** 1 Clinical Neurophysiology and Functional Neuroimaging Unit, Department of Neurological and Movement Sciences, University of Verona, Verona, Italy; 2 Department of Computer Science, University of Verona, Verona, Italy; 3 Neuroradiology Unit, Department of Diagnostic and Pathology, University Hospital Verona, Verona, Italy; 4 Department of Neurophysiology, Foundation IRCCS San Camillo Hospital, Venice, Italy; 5 Epilepsy Surgery Center, Niguarda Hospital, Milan, Italy; 6 Institute of Biomedical Engineering, University of Oxford, Oxford, United Kingdom; 7 Clinical Neurology Unit, Department of Medical Science, Surgery and Health, University of Trieste, Trieste, Italy; Medical Photonics Research Center. Hamamatsu University School of Medicine, JAPAN

## Abstract

Electrophysiological and hemodynamic data can be integrated to accurately and precisely identify the generators of abnormal electrical activity in drug-resistant focal epilepsy. Arterial Spin Labeling (ASL), a magnetic resonance imaging (MRI) technique for quantitative noninvasive measurement of cerebral blood flow (CBF), can provide a direct measure of variations in cerebral perfusion associated with the epileptic focus. In this study, we aimed to confirm the ASL diagnostic value in the identification of the epileptogenic zone, as compared to electrical source imaging (ESI) results, and to apply a template-based approach to depict statistically significant CBF alterations. Standard video-electroencephalography (EEG), high-density EEG, and ASL were performed to identify clinical seizure semiology and noninvasively localize the epileptic focus in 12 drug-resistant focal epilepsy patients. The same ASL protocol was applied to a control group of 17 healthy volunteers from which a normal perfusion template was constructed using a mixed-effect approach. CBF maps of each patient were then statistically compared to the reference template to identify perfusion alterations. Significant hypo- and hyperperfused areas were identified in all cases, showing good agreement between ASL and ESI results. Interictal hypoperfusion was observed at the site of the seizure in 10/12 patients and early postictal hyperperfusion in 2/12. The epileptic focus was correctly identified within the surgical resection margins in the 5 patients who underwent lobectomy, all of which had good postsurgical outcomes. The combined use of ESI and ASL can aid in the noninvasive evaluation of drug-resistant epileptic patients.

## Introduction

Multimodal approaches combining several imaging methods may further our understanding of the mechanisms underlying the epileptic process and aid in more accurately localizing abnormal neuronal activity, especially in patients with drug-resistant epilepsy [1–2]. In such patients, the mainstay therapeutic option for reducing or suppressing seizures is surgical resection of the epileptogenic zone [3–5]. Therefore, precise preoperative localization of the epileptogenic zone is crucial to spare non-epileptogenic brain tissue as best as possible and minimize postoperative neurological deficits [6]. Although highly invasive and burdened by potential risks limiting its use in clinical settings [7], invasive electroencephalography (EEG) (e.g., stereo-EEG [sEEG]) remains the gold standard to localize the epileptogenic focus when noninvasive presurgical evaluation fails to yield clear-cut information [8–10].

Noninvasive presurgical workup will usually include long-term EEG, video-EEG and neuropsychological testing, which do not always provide the localization accuracy and precision required for surgical planning. Morphological magnetic resonance imaging (MRI) scans are also routinely acquired in epileptic patients to identify structural brain lesions such as tumors, cortical dysplasia or hippocampal sclerosis, which can define the seizure onset zone and guide surgical resection [11]. In the absence of these findings, however, the MRI scans may be inconclusive for delineating the location of the focal abnormality and will therefore need to be integrated with complementary neuroimaging studies.

Imaging techniques such as positron-emission tomography (PET) and single-photon emission computed tomography (SPECT) [12–15], dipole localization or electrical source imaging (ESI) [16–19], and EEG-functional MRI (fMRI) [20–23] can all offer additional localization information and improve the yield of routine imaging studies. However, functional neuroimaging techniques (as PET, SPECT, fMRI), though each providing data on metabolism, perfusion, and blood oxygenation, have poor temporal resolution. Conversely, EEG, and high-density EEG (hdEEG) in particular, provide a direct measurement of neuronal activity with high temporal resolution which allows investigation of epileptic activity on a millisecond scale, from the initiation of seizure activity through to the propagation phase [24]. When combined, they can be used to evaluate the same phenomenon from different perspectives, overcoming the limitations inherent to each modality and thus obtain a more complete picture of the dynamics of the epileptic focus [25–29].

Since the 1980s, perfusion changes during epileptic processes have been investigated using SPECT in combination with technetium-99m hexamethyl-propylene amine oxime (Tc-99m HMPAO) or Tc-99m ethyl cysteinate dimer (Tc-99m ECD), especially during the ictal and postictal phases [30]. While ictal and postictal SPECT demonstrate high sensitivity in focus localization (97–100% and 75–77%, respectively), their sensitivity during the interictal phase is about 50% lower [31–32]. Moreover, the technique has several limitations, including relatively low spatial resolution, high cost, and difficult logistics that restrict its availability to a few specialized neuroimaging centers [30]. In addition, the localization accuracy of SPECT during the ictal/early postictal phases depends critically on injecting the tracer as early as possible after seizure onset. If the timing is not precise, ambiguous blood flow changes may result, leading to misinterpretation of the main generator of seizure activity.

Recently, newer MRI methods to study local cerebral perfusion have been proposed, offering several substantial advantages over nuclear medicine techniques, including noninvasiveness, no radiation exposure, easier accessibility, and higher spatial resolution [33–34]. Among these innovative techniques, Arterial Spin Labeling (ASL) MRI has been applied to noninvasively study and quantify perfusion changes related to the epileptic focus. ASL provides a quantitative measurement of regional cerebral blood flow (CBF) without the need for contrast agents. Similar in principle to nuclear medicine techniques, ASL utilizes magnetically labeled arterial blood-water proximal to the tissue of interest as an endogenous diffusible tracer [35–36]. The approach is to collect a labeled and a control image, which are equal for the static tissue signal but have different weights on flowing spins. Their subtraction (control-label) removes the signal from tissue, giving only the signal contribution from the CBF. Since the signal difference is on the order 0.5–1.5% of the full signal, the signal-to-noise (SNR) ratio in this technique is intrinsically low, so that multiple volume repetitions are needed to ensure a sufficient signal level and quantify the different perfusion parameters [37]. The noninvasive nature of ASL makes it a potential alternative to further invasive examinations with [^18^F]FDG-PET or SPECT, which are still considered the most useful neuroimaging techniques in the assessment of epileptic patients. Moreover, because it does not require the injection of radioligands, thus obviating the need to wait for tracer uptake, ASL can be usefully applied to map brain changes during the interictal, as well as the ictal and early postictal phases. To date, ASL has seldom been employed in epilepsy [38–40], but promising results have been obtained in combination and comparison with other techniques [41–42]. In a previous work, we assessed the diagnostic value of ASL perfusion MRI in identifying the epileptogenic zone by comparing the results to those obtained with ESI and [^18^F]FDG-PET in a group of 6 patients with drug-resistant focal epilepsy [29]. In all cases, the concordance between the altered patterns of metabolism and perfusion during both the interictal and early postictal phases was good. Moreover, the functional changes overlapped fairly well with the electrical changes localized by ESI, further demonstrating the usefulness of ASL as a valid alternative to [^18^F]FDG-PET.

The complexity and high variability of epilepsy (e.g., focus localization and structural, hemodynamic and metabolic abnormalities) often preclude group analyses, so that the individual peculiarities of each patient need to be assessed separately. The simplest quantitative approach to patient-specific analysis is represented by a one-versus-many parametric test, where a single patient is compared to a group of age-matched controls [43–44]. In order to perform this type of analysis and automatically identify the areas of statistically altered perfusion, two general approaches are currently in use. One is the homoscedastic approach, also referred to as the random-effect model [45–47], which assumes homogeneous within-subject variance across subjects or negligible by comparison to between-subject variance. The other is the heteroscedastic approach, also referred to as the mixed-effect model [48–49], which takes the heterogeneous within-subject variances into account. In ASL data, the importance of this second approach was recognized by Viviani et al. in 2009 [50] and then extensively investigated by Maumet and colleagues in 2013 [51], using general linear model analyses based on these two approaches in patients with brain tumors.

In this study, we used source imaging and electrophysiological data as the reference for focus localization to further assess the feasibility of ASL in detecting perfusion changes related to the epileptic focus in patients affected by drug-resistant focal epilepsy and potential candidates for surgery. Furthermore, we aimed to quantitatively detect the brain perfusion abnormalities in each patient, defining a template-based approach combined with a heteroscedastic model for the automatic detection of statistically significant CBF changes. Finally, the findings from postsurgery MRI scans and the clinical outcomes in a subgroup of patients were used as “ground truth” to evaluate the localization accuracy of the ASL and ESI results.

## Materials and Methods

### Patients and Subjects

The study population was 6 consecutive patients (hereafter, patients nos. 7–12) with drug-resistant focal epilepsy admitted for presurgical assessment according to the following criteria: (i) drug-resistant epilepsy [52]; (ii) non-localizing seizure semeiology; (iii) multifocal scalp EEG discharges; and (iv) non-conclusive standard neuroimaging findings. A series of neurophysiological examinations (video-EEG and hdEEG) were performed to define clinical seizure semiology. As part of the presurgical examination protocol, advanced neuroimaging techniques, including the ASL sequence, were also performed on a 3T MRI scanner. In a previous study on a group of 6 other epileptic patients (hereafter, patients nos. 1–6) [29], the same protocol, plus [^18^F]FDG-PET, had been applied: the results from these patients are here completed with the more recent postsurgical information (when available) and ASL analyses. All new patients were classified as having either right or left temporal lobe epilepsy on the basis of clinical and electrophysiological information. In this new group, only one patient was evaluated with invasive EEG (sEEG) prior to surgery (patient no. 7).

A control group underwent MRI evaluation using the same sequences as the patient group. The control group was composed of 17 healthy subjects (10 women, mean age 34±10 years) with no history of neurological or psychiatric disorders.

In accordance with the Declaration of Helsinki, written informed consent to take part in the study was obtained from all control subjects and patients. The study protocol was approved by the Local Ethics Committee of the University Department and Hospital of Verona. The individuals in this manuscript have given written informed consent (as outlined in PLOS consent form) to publish these case details.

#### Patient No. 7

This 46-year-old, right-handed woman reported, at the age of 20, experiencing several brief episodes characterized by abnormal hearing followed by loss of consciousness on one occasion, after which she was discharged from the hospital with a diagnosis of partial epilepsy. Since then, she has had no more auditory symptoms, later classified as auras, despite having 5–6 seizures per month that she describes as sudden moments during which she cannot speak but is aware of her environment. After an attack, however, she often finds herself on the floor, unaware that she has fallen. Witnesses recalled right-sided mouth deviation, right-hand fiddling, and dystonic posturing of the left hand, followed by generalised convulsions. Standard EEG showed runs of sharp waves and rarer slow waves over the right frontotemporal leads. The MRI scan revealed no morphological abnormalities. The sEEG, subsequent to other imaging exams (hdEEG and ASL), pointed to an epileptogenic zone located over the right temporal pole, mainly involving the superior (T1) and middle (T2) temporal gyri. The patient underwent surgical removal of these portions, and has been seizure-free ever since (7 months).

#### Patient No. 8

This 62-year-old, right-handed woman suffered repeated episodes of febrile convulsions at the age of 23 months. She remained seizure-free until age 18 years, when she began experiencing brief, sudden episodes of loss of contact that went unnoticed at that time. At the age of 32, she presented a generalized seizure, and a diagnosis of epilepsy was made. Currently, she has 3–4 seizures per month characterized by oral automatism, right-hand fiddling, and left dystonic hand posturing. On repeated occasions, relatives reported brief episodes of incongruous activity, during which she is unresponsive. Standard EEG showed runs of fast, spiky activity over the frontotemporal right leads, sometimes with bilateral expression over the frontal areas. Repeated MRI scans were reportedly normal.

#### Patient No. 9

This 68-year-old, right-handed woman began suffering, at the age of 20, brief, sudden episodes of flushing, sensation of something rising from the stomach to the head followed by intense asthenia. The episodes clustered during a few months every year, then disappeared at the age of 35. Around age 60, they started again, this time associated with pervasive fear: she was initially referred for psychiatric evaluation because of suspected panic attacks. Standard EEG revealed right frontotemporal spikes, and a diagnosis of epilepsy was made. Fluid attenuated inversion recovery (FLAIR) MRI images showed bilateral hippocampal hyperintensity that was more evident on the right side.

#### Patient No. 10

This 48-year-old, right-handed man suffered febrile convulsion episodes at the age of 2 years. At the age of 18, after a secondary generalized seizure, a diagnosis of temporal epilepsy was made. With hindsight, he referred always having had brief episodes of altered sensation associated with something rising from the stomach; witnesses also reported oral automatism. Since then, he has continued to experience weekly seizures despite appropriate drug treatment. Standard EEG showed the presence of sub-continuous spiky activity over the frontotemporal left leads. The MRI scan revealed no morphological abnormalities. The patient has been seizure-free since surgical resection of the left temporal lobe (1 year ago).

#### Patient No. 11

This 48-year-old, right-handed man reported having suffered during childhood brief and sudden episodes of nausea misinterpreted as gastric disturbances at that time. At the age of 30, during hospitalization for injuries sustained in a car accident, a diagnosis of focal epilepsy was made based on EEG data and history. A few partial seizures occurred over the following months, then remitted for 15 years while he was on carbamazepine. At the age of 45, seizures reoccurred weekly, characterized by a rising gastric sensation, tachycardia, and subsequent depersonalization. Witnessed episodes were described as the patient fiddling with his right hand, dystonic posturing of the left arm, oral automatism, and inability to speak. Standard EEG showed only rare anterior temporal spikes, with right predominance. FLAIR MRI images showed mild bilateral hippocampal hyperintensity.

#### Patient No. 12

This 28-year-old, right-handed woman had a positive family history for generalized epilepsy (maternal aunt). At the age of 26, she began experiencing repeated déjà vu episodes and uncomfortable feelings sometimes associated with anxiety and tachycardia. She was unaware of subsequent vocalizations, left head version and bilateral hand fiddling, and referred suffering headaches after the symptoms resolved. Antiepileptic treatment was only partially effective. Standard EEG showed left frontotemporal spikes. MRI scans revealed altered signal areas in the right lateral frontobasal lobe, left anteroinferior frontal lobe, medio-posterior portion of the left hippocampus, and at the left frontoparietal junction.

### Data acquisition: hdEEG recording

HdEEG was performed using 256 channels (Electrical Geodesic, Inc., Eugene, OR). The net was adjusted so that Fpz, Cz, Oz, and the pre-auricular points were correctly placed according to the international 10/20 system. The net's geodesic tension structure is such that all electrodes could be evenly distributed over the scalp at approximately the same location in all patients. The data were recorded against a vertex electrode reference (Cz) at a sampling rate of 250 Hz. Patients were seated in a relaxed position; the total EEG recording time was around 40 min.

### Data acquisition: anatomical and ASL recording

The experiment was performed using a 3T MRI scanner (Allegra, Siemens, Erlangen, Germany) with a standard transmit/receive head coil. For the ASL data, a pulsed PICORE sequence (proximal inversion with a control for off-resonance effects) with the Q2TIPS scheme (QUIPSS II with thin-slice TI1 periodic saturation) was used [53]. Interleaved control and label images (80 volumes) were acquired using a 2D gradient-echo echo-planar imaging (GRE-EPI) readout with the following scan parameters: TR/TE = 3500/16 ms; TI_1_/TI_s_/TI_2_ = 700/1400/1600 ms and 90° flip angle. Sixteen axial slices in ascending order were prescribed and positioned parallel to the anterior-posterior commissure line (3.5x3.5x5 mm^3^, with an inter-slice gap of 1 mm) in order to cover the presumed focus location. The labeling slice was 10 cm thick and was separated from the proximal slice of the acquisition volume by a 20-mm gap. A calibration scan with the same parameters as the ASL sequence but longer TR (10 s) was also acquired to estimate the equilibrium magnetization of arterial blood (M_0b_). The overall acquisition time for ASL was approximatively 6 mins, including the additional calibration scan. A high-resolution whole brain anatomical scan was acquired for each subject using a 3D T1-weighted magnetization prepared rapid acquisition gradient echo sequence ([MPRAGE], TR/TE = 2300/3.9 ms; FOV = 192 x 192; matrix = 256 x 256; 176 sagittal slices 1.0 mm in thickness). Other high-resolution anatomical images, including T2-weighted, FLAIR and inversion recovery with 2D readout, were also acquired in order to assess for the presence of structural alterations such as hippocampal sclerosis or malformations of cortical development.

### Data analysis: electrical source imaging

HdEEG data were analyzed using Cartool software (http://sites.google.com/site/cartoolcommunity/). The T1-weighted anatomical images were used to create a realistic model of the brain for source localization. In each patient, the hdEEG and MRI data and the solution space were restricted to the gray matter. The solution space for the distributed source model contained from 3,028 to 3,088 points uniformly distributed over the gray matter of the brain and mapped onto the spherical head model with anatomical constraints (SMAC) space [54]. The peak of the spike was used as a trigger for averaging in epochs of ±500 ms. Two time frames were chosen to characterize spike topography: the first, from the beginning of the spike to the time point at 50% of the rising phase, was defined as an epoch characterizing a possible source of the spike generator [24]; in the second, an epoch at the peak of the spike was defined as indicating propagation. A standardized source imaging procedure, ([LORETA] low resolution brain electromagnetic tomography) [55] constrained to the individual gray matter, was applied to the averaged spikes. From the ESI, the current density (CD) was quantified at each solution point [μA/mm^3^].

### Data analysis: ASL quantification

ASL data were preprocessed and analyzed using FSL 5.0.1 (FMRIB, Oxford, UK) and Matlab 7.14 (MathWorks, Natick, MA) with a dedicated home-made code created for this study. Motion correction was applied separately to the Control and Label volumes using the MCFLIRT tool and taking the first volume as reference. In particular, a six-parameter 3D rigid-body registration with a normalized correlation cost function was used. The ASL calibration scan was used for estimating the coregistration parameters from ASL to the individual T1-weighted image by applying a 3D rigid-body registration with a normalized mutual-information cost function and 7 degrees of freedom.

After these pre-processing steps, surround subtraction was applied to the Control and Label volumes to obtain a 4D matrix representing the perfusion-weighted maps. Unlike what is usually done in ASL studies, ΔM represents the whole set of perfusion-weighted maps (one volume per repetition) instead of a single perfusion-weighted map obtained by averaging across the repetitions. In this way, a 4D matrix for the CBF parameter can be estimated for each subject and the temporal information given by the different repetitions will allow measurement of the within-subject variance [51]. The standard kinetic model [56] was applied to the 4D ΔM matrix to estimate the blood flow maps in physiological units [ml/100g/min]. The CBF values were calculated as follows:

$$CBF\;=\;\frac{\Delta M}{2\alpha M_{ob}{TI}_{1}e^{\frac{-({TI}_{2}+\left(n-1\right)*{slice}_{time})}{T_{1B}}}}$$

where *ΔM* is the difference signal, *TI*_*1*_ and *TI*_*2*_ are the sequence time parameters described above, *n* is the slice number, *slice*_*time*_ is the time taken to acquire each single slice (~ 50ms), *T*_*1B*_ is the longitudinal relaxation time of blood (1664 ms at 3T), and *α* is the inversion efficiency (0.95 for pulsed ASL) [57]. *M*_*ob*_ was calculated as the ratio between the mean tissue equilibrium magnetization value in a cerebrospinal fluid (CSF) region (from the calibration scan) and the brain-blood partition coefficient.

For each subject, the mean and variance values over all repetitions were calculated for the CBF parameter. These maps in ASL space were affine-registered to the individual high-resolution anatomical images by applying the previously estimated transformation matrix. Each T1-weighted image was then registered to the Montreal Neurological Institute (MNI) space with 1x1x1 mm^3^ resolution using a non-linear method (FNIRT tool in FSL). Finally, the joint ASL/T1-weighted and T1-weighted/MNI space transformation parameters were used to spatially normalize the CBF maps representing the mean and variance values.

### Data analysis: patient-specific detection of perfusion changes

For each subject, the CBF images in MNI space were intensity normalized to compensate for mean inter-subject perfusion variations. Indeed, voxel-wise ASL detection studies, where the focus is on local variations across the brain, have shown that an intensity normalization step is advised to increase the sensitivity [58], also due to the large inter-subject variability in global CBF values [59]. The value used for the intensity normalization was equal to the mean CBF in gray matter, applying a threshold of 70% for detecting pure gray matter voxels, similarly to [51, 60]. The partial volume estimates for gray matter were derived from segmentation of the normalized anatomical image using the FAST tool in FSL. In addition, the non-physiological negative perfusion estimates present in the gray matter normalization mask were excluded from the mean CBF calculation in order to define a more reliable reference value [51, 60].

For comparing each patient to the control group, a two-step procedure was performed: construction of a normal perfusion template and then a one-versus-many statistical analysis. The heteroscedastic approach was used in the analysis (mixed-effect model) [46, 50–51].

The first step entailed CBF template construction from the control group images. The template was created using the FLAME 1 (FMRIB's local analysis of mixed effects-stage 1) tool available in FSL software [61–62], which uses Bayesian modeling and explicitly accounts for the within-subject variance. For each subject, normalized CBF images representing the mean CBF estimates and the CBF within-subject variance were supplied to the FLAME 1 tool. The data from the healthy subjects were pooled into one group, and the group maps (mean and variance) were estimated with this tool. In a heteroscedastic model, the group variance will reflect both the within-subject and the between-subject variability.

In the second step, comparison of the control group versus each single patient was carried out using an unpooled-variance unpaired two-sample *t*-test in order to consider both the group variance and the patient variance. The *t*-statistic, for testing whether the estimated patient versus control group means were different, was calculated as:

$$t\;=\;\frac{\hat{\mu_{c}}-\;\hat{\mu_{p}}}{\sqrt{\frac{\hat{\sigma_{c}^{2}}}{N_{c}}+\frac{\hat{\sigma_{p}^{2}}}{N_{p}}}}$$

where *μ* and *σ* ^*2*^ represent the estimated mean and variance for the patient (*p*) and the control group (*c*) respectively, and *N* represents the number of subjects (17 and 1 for *N*_*c*_ and *N*_*p*_, respectively). A negative *t*value indicates that, at the voxel of interest, the mean is higher in the patient than in the control group, thus identifying possibly hyperperfused areas (patient > controls). Conversely, a positive *t*value indicates possible areas of hypoperfusion (patient < controls).

The simplest approximation was adopted for defining the degrees of freedom (df), assigning *df = n-p-1*, where *n* is the total number of subjects and *p* the number of fixed (dummy) variables such as subject-specific covariates like age or behavioral data [49]. The same approach was also implemented in the statistic test in FLAME 1 of FSL [62]. A false discovery rate (FDR) correction (*q* < 0.05) for multiple comparisons was applied to the statistical results [63] to detect the areas of statistically significant alterations in perfusion.

The procedure for the automatic detection of patient-specific perfusion abnormalities was applied to the 6 new patients and to the 6 patients evaluated in the previous study in order to further confirm the results and the observations from the region of interest (ROI) analysis.

### Data analysis: ESI and ASL comparison

For each patient, the mean CBF map from ASL and ESI results was overlaid on individual T1-weighted anatomical MRI images to combine all of them in the same anatomical space using FSL tools. As in the previous study [29], a series of ROIs was identified using the Harvard-Oxford Atlas to compare the ESI and ASL results in terms of CD and CBF inside the same areas, using the spatially normalized data. Quantification was restricted to the gray matter, including voxels whose probability to belong to the gray matter was at least 80%. All measures were compared to the contralateral side. The five ROIs with the highest differences in CD and CBF values are reported here. This was done to quantitatively compare the information provided by ASL and ESI and to evaluate the agreement with the results of the statistical analysis. Areas over the epileptic focus are expected to be characterised by high CD values and low CBF values in comparison to the correspondent contralateral ROI, if the patient is in interictal phase.

### Surgical validation

In patients undergoing surgical resection, freedom from seizure following the operation can be considered the ground truth and the most unambiguous proof of correct localization of the epileptogenic focus [18]. In order to assess the localization reliability of the ESI and ASL results, the presurgical information were overlaid on the postoperative MRI scan of each patient to determine whether or not the ESI and ASL maps were congruent and overlapped well with the resected tissue areas. Importantly, although the extension of a resected area tends to be overestimated and larger than the actual epileptogenic zone in most patients, seizure freedom following resection can be taken as proof of correct localization on a sublobar level [64]. Therefore, the preoperative anatomical MRI scan was coregistered with the postoperative scan, and the estimated coregistration parameters were inversely transformed in order to project the postoperative image over the original preoperative scan. To assess the ESI and ASL results, the rising phase of epileptiform activity and the statistical maps were used, respectively, and were overlapped on the coregistered postoperative anatomical image.

## Results

**Fig 1:**
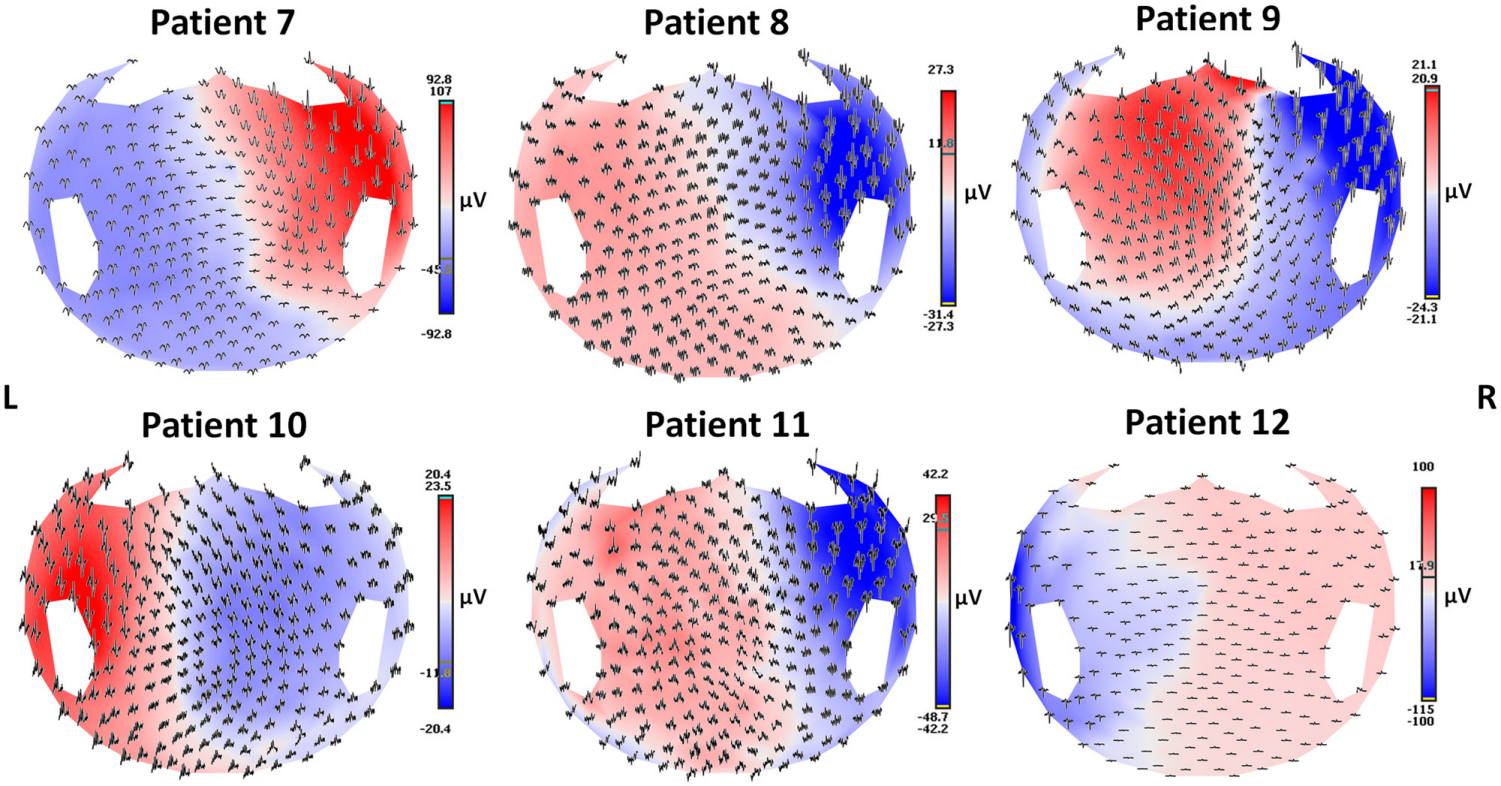
Average spike voltage maps. Spike average (epochs of 1 s) of interictal activities visualized according to the projected location of the scalp electrodes in the six patients.

**Fig 2:**
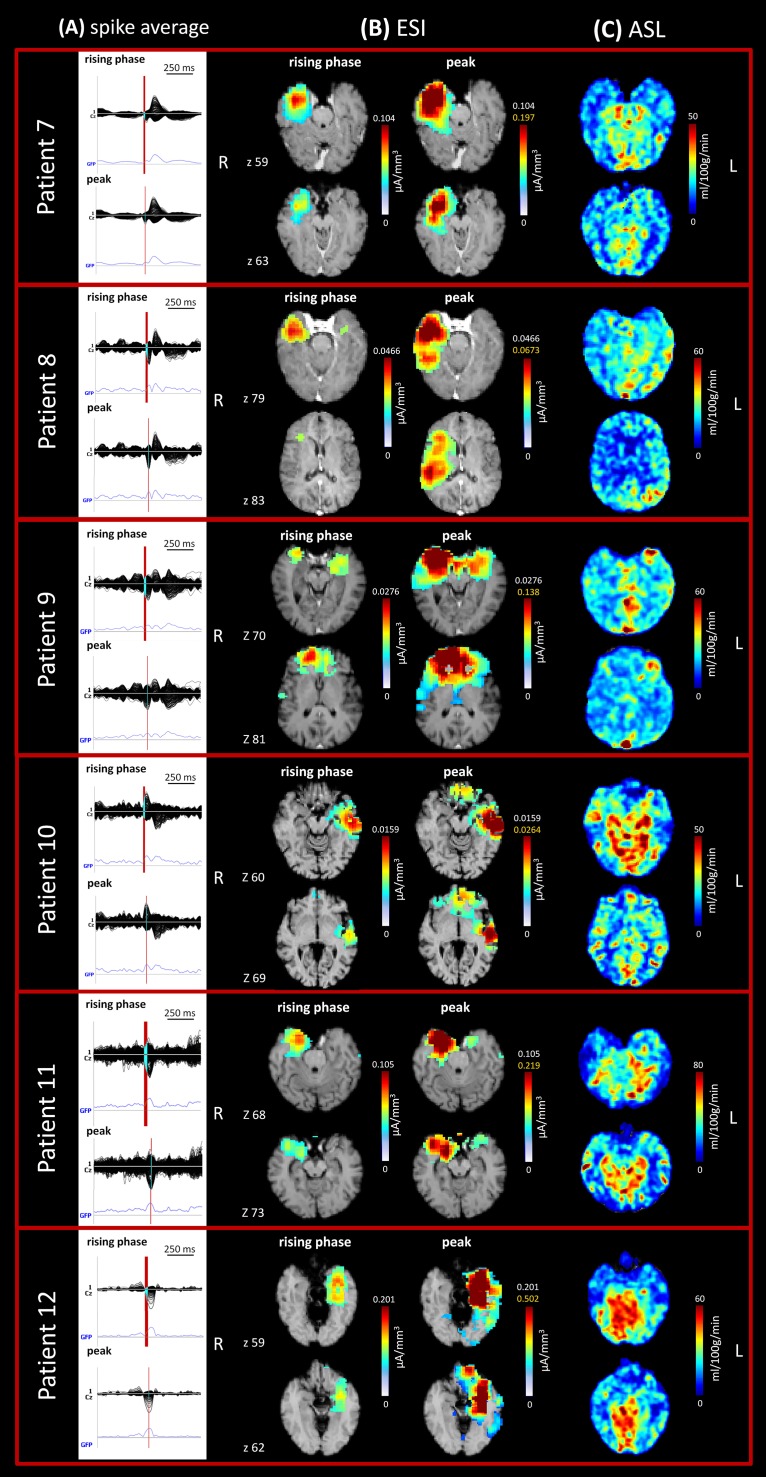
Imaging analysis results in the six patients (patients nos. 7–12) with focal epilepsy. ESI and ASL images of the same anatomical space were acquired for each patient and two axial sections are shown (z coordinates in native space). (A) Spike average: 256-channel EEG traces with a duration of 1 s (spike average). The global field power is used for the onset (red line). (B) ESI results: EEG source imaging at 50% rising phase of the peak (up) and at the peak (down). The scale indicates the current density (CD) [μA/mm^3^]. (C) ASL results. The scale indicates the cerebral blood flow (CBF) values [ml/100g/min].

All 6 patients (patients nos. 7–12) in the new group completed the protocol. Clinical information and quantification results for each patient are presented in Tables 1 and 2 and Figs 1 and 2.

**Table 1:** Clinical profile: age, gender, MRI abnormalities, EEG activity, and location of noninvasive techniques (ESI and ASL) and invasive techniques (sEEG).

Pt	Sex	Age	Years since beginning	Seizure frequency	Hx	Current antiepileptic therapy	Standard MRI	EEG	ESI	ASL	sEEG
7	F	46	26	5–6/month	negative	VPA, LEV, ZNS	negative	sharp-waves over the frontotemporal right derivations	localization in right anterior temporal areas	hypoperfusion in right temporal and frontotemporal regions	right temporal pole focus
8	F	62	30	3–4/month	febrile convulsion	CBZ	negative	runs of fast, spiky activity over the frontotemporal right leads	localization in right temporal and frontotemporal areas	hypoperfusion in right temporal and frontotemporal regions	none
9	F	68	48	1/day	negative	CBZ, LTG	bilateral hippocampal hyperintensity	right frontotemporal spikes	localization in right temporal and frontotemporal regions	hypoperfusion in right temporal regions	none
10	M	48	30	1/day	febrile convulsion	CBZ, LEV	negative	left frontotemporal spikes	localization in left anterior temporal areas	hypoperfusion in left temporal and frontotemporal regions	none
11	M	48	18	1/week	negative	VPA, ZNS, LTG	mild bilateral hippocampal hyperintensity	rare anterior temporal spikes, with right predominance	localization in right anterior temporal areas	hypoperfusion localized to right temporal regions	none
12	F	28	2	2–3/month	familiar	CBZ, VPA	altered areas in the right lateral frontobasal lobe, left anteroinferior frontal lobe, medioposterior portion of the left hippocampus, left frontoparietal junction	left frontotemporal spikes	localization in left temporal and frontotemporal regions	hypoperfusion localized to left temporal regions	none

VPA = valproate; LEV = levetiracetam; ZNS = zonisamide; CBZ = carbamazepine; LTG = lamotrigine.

**Table 2:** Quantification results from ESI and ASL in the six patients (patients nos. 7–12) with focal epilepsy.

Regions of Interest										
**Subject 7**	**r-H**	**l-H**	**r- ITGa**	**l- ITGa**	**r-TP**	**l-TP**	**r-FOrC**	**l-FOrC**	**r-TFCa**	**l-TFCa**
**CD**	0.047	0.009	0.123	0.010	0.066	0.013	0.037	0.015	0.142	0.012
**CBF**	27.45	38.44	28.73	35.22	26.47	39.36	20.66	35.72	29.81	36.18
**Subject 8**	**r-MTGa**	**l-MTGa**	**r- ITGp**	**l- ITGp**	**r-FoC**	**l-FoC**	**r-CoC**	**l-CoC**	**r-PP**	**l-PP**
**CD**	0.033	0.018	0.032	0.019	0.025	0.009	0.019	0.008	0.019	0.006
**CBF**	31.97	39.76	32.12	45.08	32.03	38.43	33.08	37.95	36.27	44.44
**Subject 9**	**r-TP**	**l-TP**	**r-MTGa**	**l-MTGa**	**r-STGa**	**l-STGa**	**r-FP**	**l-FP**	**r-FOrC**	**l-FOrC**
**CD**	0.045	0.023	0.032	0.020	0.038	0.017	0.044	0.022	0.040	0.016
**CBF**	38.03	45.48	38.42	43.60	31.12	39.26	35.52	37.17	37.90	47.51
**Subject 10**	**r-TP**	**l-TP**	**r-MTGp**	**l-MTGp**	**r-TFCa**	**l-TFCa**	**r-STGa**	**l-STGa**	**r-FOrC**	**l-FOrC**
**CD**	0.006	0.012	0.003	0.010	0.006	0.012	0.003	0.010	0.003	0.010
**CBF**	32.50	23.63	35.60	27.35	36.50	31.30	49.10	38.30	46.25	35.62
**Subject 11**	**r-TP**	**l-TP**	**r-ITGa**	**l-ITGa**	**r-ITGp**	**l-ITGp**	**r-STGa**	**l-STGa**	**r-TFCa**	**l-TFCa**
**CD**	0.087	0.047	0.093	0.034	0.051	0.035	0.033	0.039	0.143	0.047
**CBF**	40.23	45.62	22.88	29.89	32.81	37.25	47.20	55.32	20.45	26.18
**Subject 12**	**r-TP**	**l-TP**	**r-ITGa**	**l-ITGa**	**r-MTGp**	**l-MTGp**	**r-H**	**l-H**	**r-TFCa**	**l-TFCa**
**CD**	0.123	0.227	0.060	0.196	0.066	0.236	0.050	0.094	0.102	0.224
**CBF**	37.20	26.35	40.32	30.35	45.87	31.58	49.10	38.15	32.05	29.24

ESI indicates current density (CD) at 50% rising phase of the peak [μA/mm^3^], while ASL indicates cerebral blood flow (CBF) [ml/100g/min]. Only the values for the five most significant regions are reported. H = hippocampus; ITGa = inferior temporal gyrus, anterior division; TP = temporal pole; FOrC = frontal orbital cortex; TFCa = temporal fusiform cortex, anterior division; MTGa = middle temporal gyrus, anterior division; ITGp = inferior temporal gyrus, posterior part; FoC = frontal opercolum cortex; CoC = central opercolar cortex; PP = planum temporale; STGa = superior temporal gyrus, anterior division; FP = frontal pole; MTGp = middle temporal gyrus, posterior division.

On hdEEG, focal source localization was detected in all patients. Source analysis reliably revealed the area of initial epileptic activity (at 50% rising phase of interictal epileptiform discharges [IEDs]) [24] and the presumed epileptogenic zone in all patients. All patients were evaluated during the interictal phase and all had temporal seizures, with abnormal activity localized mainly over the temporal area (or more anterior temporal areas) which spread to the frontotemporal area at the peak of activity in some cases.

**Fig 3:**
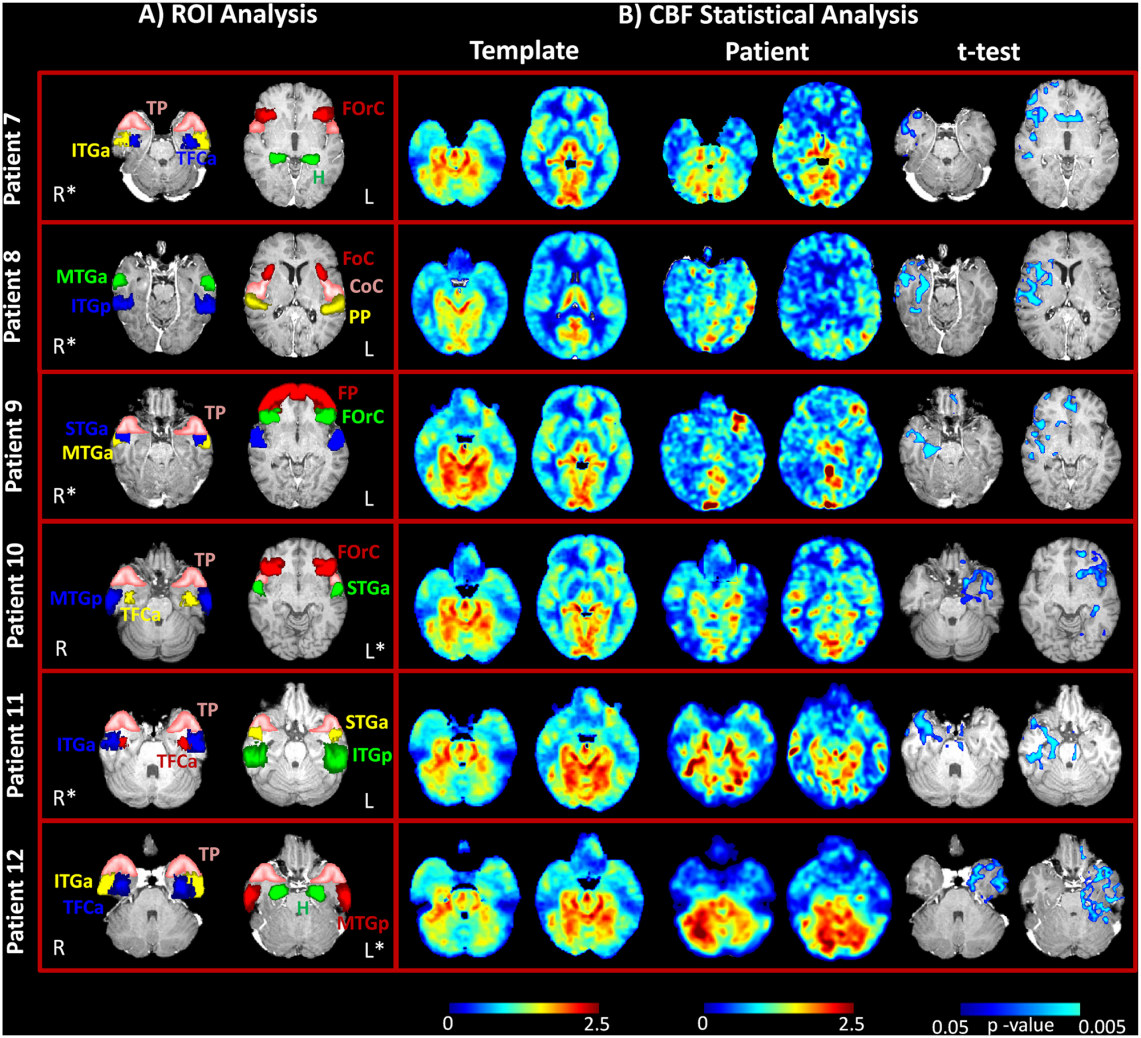
ASL imaging results in the new group of six patients (patients nos. 7–12) with focal epilepsy. A) Regions of interest (ROIs) for the quantification of current density (CD) and cerebral blood flow (CBF) values at the same anatomical level in each subject. ROIs are superimposed over axial T1-weighted slices in MNI space. B) Statistical analysis results from the template-based comparison. CBF maps (normalized values) for the same axial slices reported in part A) are shown here for the control group (template) and patients, together with the statistical map. In all patients, only areas with a statistically significant decrease in perfusion as compared to the healthy subjects were detected (blue scale, FDR corrected, *q* < 0.05).

**Fig 4:**
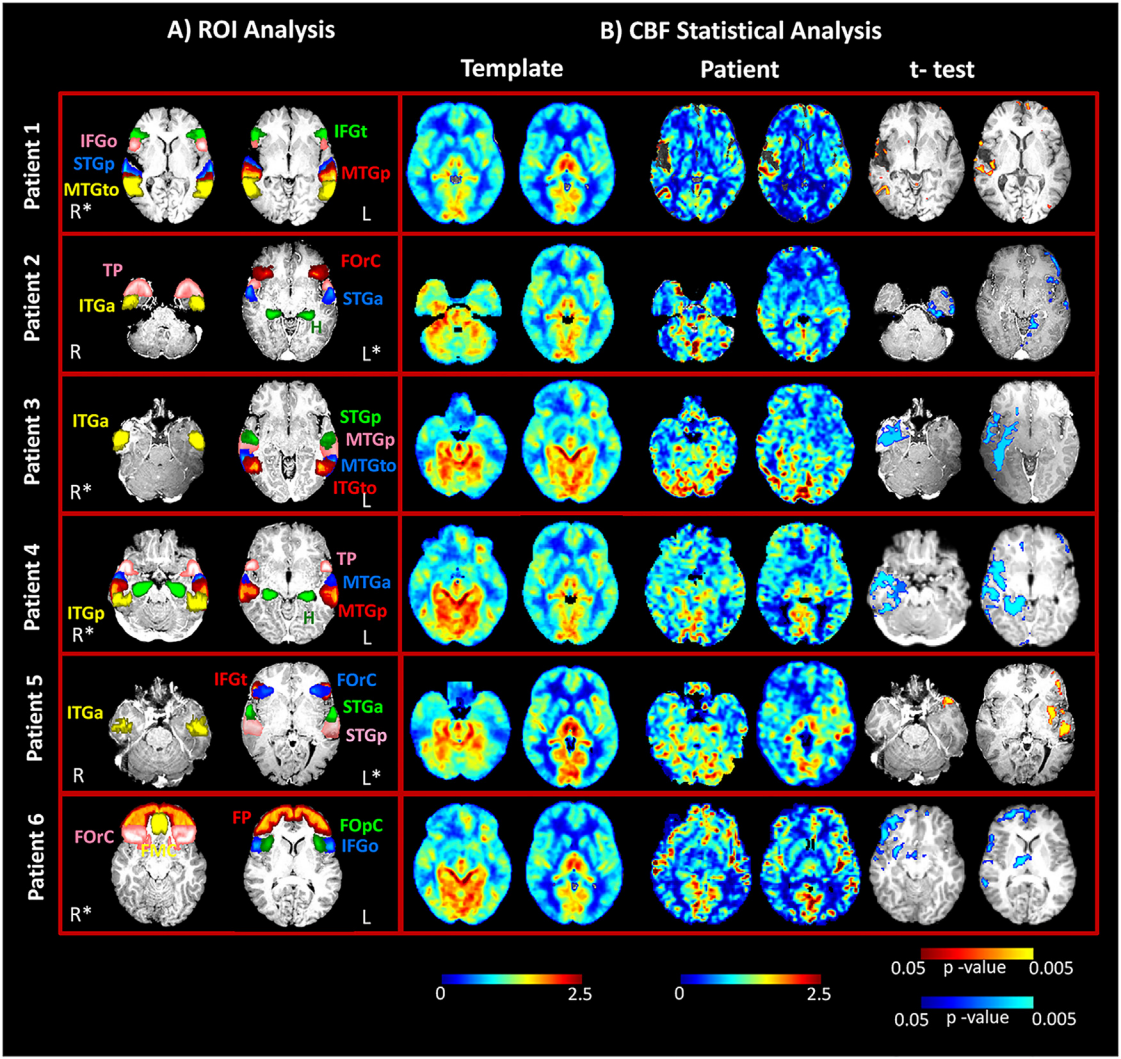
ASL imaging results in the six focal epilepsy patients described in the previous study (patients nos. 1–6). A) Regions of interest for quantification of current density (CD) and cerebral blood flow (CBF) values at the same anatomical level in each subject. ROIs are superimposed over axial T1-weighted slices in MNI space. B) Statistical analysis results from the template-based comparison. CBF maps (normalized values) for the same axial slices reported in part A) are shown here for the control group (template) and patients, together with the statistical map. Areas with a statistically significant decrease in perfusion (hypoperfusion), as compared to the healthy subjects, were detected in four patients (blue scale, FDR corrected, *q* < 0.05). Conversely, areas with a statistically significant increase in perfusion (hyperperfusion), as compared to the healthy subjects, were detected in two patients (yellow scale, FDR corrected, *q* < 0.05).

ASL changes were observed in all patients, and these activities were concordant with the source results. Hypoperfusion patterns were identified during interictal activity in all 6 patients. Statistical analysis automatically identified the areas of significantly decreased perfusion in all patients as compared to the control group and further confirmed the results of the ROI analysis, even in those patients in which the hemispheric asymmetries were not marked and the CBF maps were difficult to interpret (patients nos. 9 and 12). Fig 3 illustrates the results from the statistical analysis of the 6 new patients, together with the localization of the ROIs in each patient. Fig 4 shows the same information for the group of 6 epileptic patients (patients nos. 1–6) described in our previous study. In this case, hypoperfused areas were identified during the interictal phase (4 out of 6 patients), while hyperperfusion was detected in the postictal period (2 out of 6 patients).

**Fig 5:**
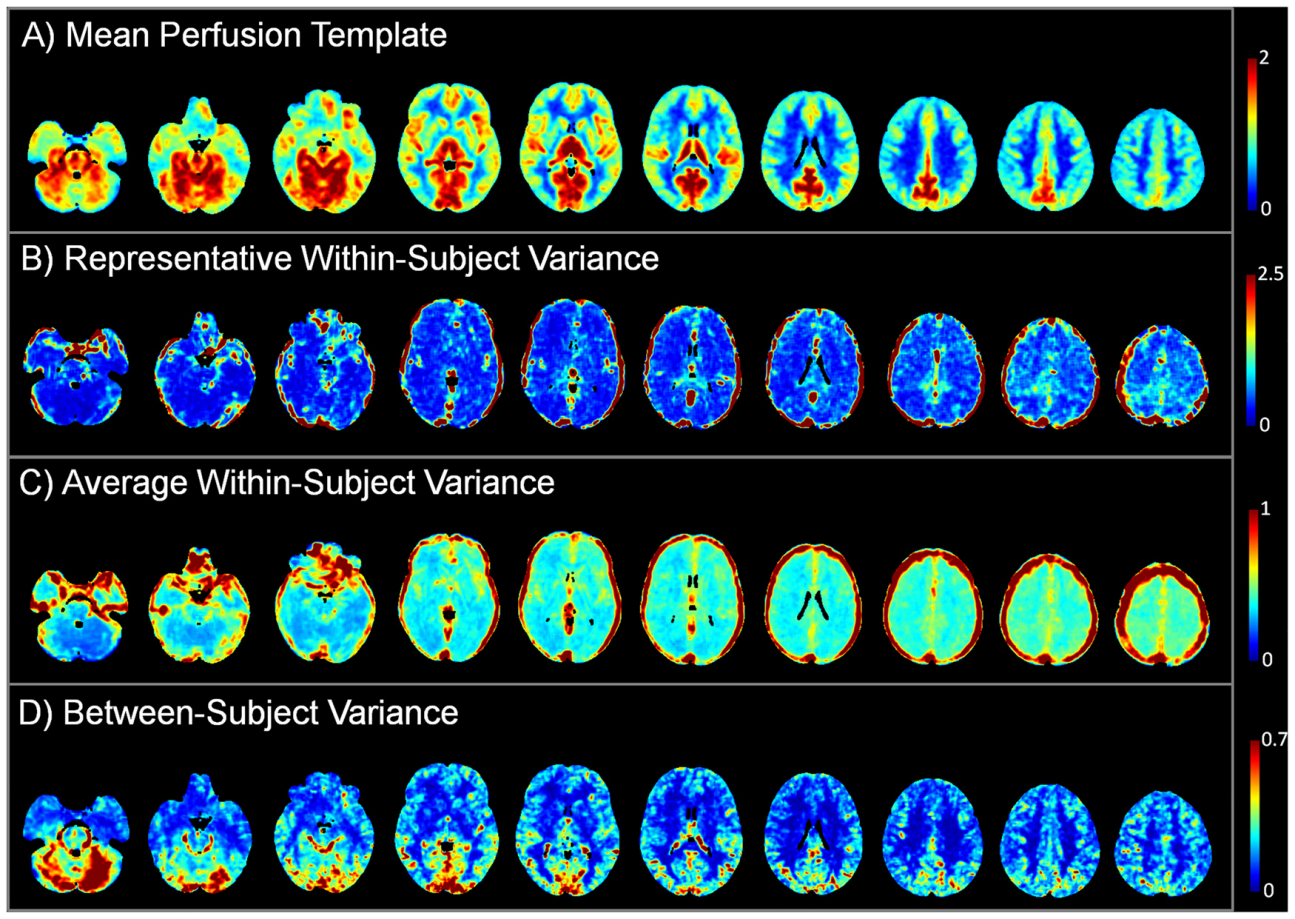
Parameter estimates for the normal perfusion template, computed from the control group. A) Mean CBF estimates expressed in normalized units. B) Within-subject variance, estimated from the temporal information given by the multiple repetitions, for a representative subject. C) Average within-subject variance in the control group. D) Between-subject variance estimated from the heteroscedastic model. Axial slices of interest are displayed in radiological convention.

Several sections of the normal perfusion template employed in the statistical analysis comparison are reported in Fig 5 in order to provide additional information for better interpreting each individual case. This figure includes the mean perfusion estimates (A) and the between-subject variance (D), as estimated by the heteroscedastic model from the data of the control group. In addition, we reported for a representative subject the within-subject variance (B), which was estimated across the different repetitions, and the average within-subject variance across all the subjects of the control group (C), to provide a global measure of the expected within-subject variance. Interestingly, the vascular structures are characterized by high variance values, in agreement with previous findings [50–51].

### Single-subject results

#### Patient No. 7

The hdEEG showed runs of spikes over the right anterior temporal derivations; this pattern was associated with rare asynchronous spikes over the contralateral frontotemporal areas. On 2D visualization, they localized on the right anterior zygomatic leads (Fig 1). The CBF maps showed a reduction in perfusion in the right temporal regions (temporal pole [TP], inferior temporal gyrus—anterior division [ITGa], and hippocampus [H]) and in some parts of the right frontal lobe, mainly the frontal operculum cortex (FOrC) (Table 2; Figs 2 and 3a). These results were in agreement with source imaging, which placed the rising phase of the activity in the right temporal regions (mainly TP, ITGa, and temporal fusiform cortex—anterior division [TFCa]). Propagation of activity also remained restricted to those regions (Table 2; Fig 2). The ASL results from the ROI analysis were further confirmed by the statistical analysis, which detected statistically significant areas of hypoperfusion over the r-TP, r-ITGa, and r-FOrC in comparison to the control group (FDR, *q* < 0.05). The alterations over the hippocampal structures did not reach statistical significance, however (Fig 3b).

#### Patient No. 8

The hdEEG showed spiky activity over the right temporal leads; this pattern was rarely associated with spreading to the ipsilateral frontal leads. On 2D visualization, the activity localized over the right zygomatic area (Fig 1). The CBF maps showed hypoperfusion in the right temporal areas (inferior temporal gyrus—posterior division [ITGp], middle temporal gyrus—anterior division [MTGa], and the planum temporale [PP]) (Table 2, Figs 2 and 3a). This was in agreement with the source imaging data, which placed the rising phase of activity in the right temporal areas. A marked spread around the rising localization was visible at the peak of activity (Table 2; Fig 2). Statistical analysis of the CBF maps confirmed the qualitative and quantitative results of the ROI analysis, detecting areas with a statistically significant reduction in perfusion (FDR, *q*<0.05) in the r-ITGp, r-MTGa, and frontotemporal regions (r-PP and right central opercolar cortex [r-CoC]) (Fig 3b).

#### Patient No. 9

The hdEEG yielded repeated spikes over the right anterior temporal derivations. The 2D visualization showed right anterior temporal (zygomatic leads) localization (Fig 1). The CBF maps showed mild hypoperfusion in the right temporal regions (TP, MTGa, and superior temporal gyrus—anterior division [STGa]) and FOrC (Table 2, Figs 2 and 3a). Despite the slight reduction in perfusion visible on the CBF maps and the minor differences with respect to the contralateral regions, areas of statistically significant hyoperfusion were detected by the statistical analysis (FDR, *q* < 0.05). These mainly involved the r-MTGa, r-STGa, and r-H, with spread to the right frontal areas (frontal pole [FP] and FOrC) (Fig 3b). These results were in agreement with source imaging, which placed the rising phase of activity in the right frontotemporal areas and indicated maximum activity principally in the r-FP, r-FOrC, r-MTGa, and r-STGa. Marked spread around the rising localization was visible at the peak of the spike average, partially involving the contralateral lobe (Table 2, Fig 3).

#### Patient No. 10

The hdEEG revealed repeated spikes over the left temporal derivations. The 2D rendering localized abnormal activity over the left zygomatic leads (Fig 1). Source analysis indicated maximum activity in the left temporal regions, principally localized in the TP, middle temporal gyrus—posterior division (MTGp), STGa, and TFCa. Propagation of activity was well limited to the left temporal regions (Table 2, Fig 2). The CBF maps showed areas of decreased perfusion in comparison to the contralateral side in the same left temporal regions identified by the ESI analysis and a mild decrease in the more anterior areas (Table 2, Figs 2 and 3a). This was confirmed on statistical analysis, which depicted altered hypoperfused voxels in the left temporal regions, with spreading over the frontotemporal lobe (FDR, *q* < 0.05) (Fig 3b).

#### Patient No. 11

The hdEEG showed rare spike waves over the right temporal derivations. The 2D rendering localized these abnormalities over the right zygomatic temporal leads (Fig 1). Source analysis indicated maximum activity in the right temporal regions, principally localised in TP, ITGa, ITGp, and STGa. Propagation of activity remained well restricted to the rising localization (Table 2, Fig 2). Decrease in CBF was visible in the right temporal regions as compared to the left ones (Table 2, Figs 2 and 3a), which reached statistically significance (FDR, *q* < 0.05) mainly over the right TP, ITGp, and STGa (Fig 3b).

#### Patient No. 12

The hdEEG showed spike-wave activity and slowing over the left temporal derivations. The 2D rendering localized the epileptiform abnormalities over the left temporal leads (Fig 1). Source analysis indicated maximum activity over the left temporal regions, principally localised in the TP, ITGa, MTGp, and TFCa. Mild propagation of activity was present around the rising localization and more anteriorly (Table 2, Fig 2). The CBF maps showed a slight reduction in perfusion over the left temporal lobe (Table 2, Figs 2 and 3a), which was confirmed by statistical analysis (FDR, *q*<0.05) showing involvement of all left temporal areas (Fig 3b).

### Statistical analysis of the previously studied epileptic group

Statistical analysis based on the normal perfusion template was also applied to the previously studied group of 6 epileptic patients in order to evaluate agreement with the ROI analysis and the obtained results (Fig 4). In the two patients evaluated in the postictal phase (patients nos. 1 and 5), the areas of hyperperfusion previously depicted by the ROI analysis were confirmed by the statistic, which showed areas of significantly increased perfusion. Significant voxels were mainly located perilesionally in patient no. 1 and mainly distributed over the left STG and more anteriorly in patient no. 5, both findings in agreement with the ROI analysis. In patient no. 2, areas of significantly reduced perfusion were detected over the left TP, ITGa, and H, confirming the visual and quantitative analysis with the ROI approach. Large areas of significant hypoperfusion were identified over the right temporal lobe in two patients (nos. 3 and 4). In particular, in the case of patient no. 4, the statistical analysis allowed better interpretation and identification of the altered areas on the CBF maps. Specifically, visual assessment revealed no areas of evident perfusion alteration and the ROI analysis showed only minor asymmetries between the hemispheres which were difficult to interpret, whereas statistical analysis confirmed the ROI results, clearly identifying the areas of hypoperfusion. Finally, significant areas of hypoperfusion were found mainly over the right frontal lobe in patient no. 6, particularly in the frontomesial area, confirming the visual interpretation of the CBF maps and the ROI analysis.

### Surgical Validation

Five of the 12 patients (patients nos. 2, 4, 6, 7, and 10) underwent surgical resection and 1 patient (patient no. 3) underwent brain thermocoagulation on the basis of the presurgical reported data. Besides patient no. 3, two other patients (patients nos. 6 and 7) underwent exploratory sEEG prior to surgery.

The surgical outcome in patients nos. 2 and 5 was reported in [29]; at that time, patients nos. 3 and 4 were waiting to be operated on. Patient no. 3 underwent radiofrequency thermocoagulation during a sEEG investigation to sever the right T1, hippocampus, and operculum. The patient is currently seizure free (10 months). In patient no. 4, the postoperative follow-up showed no seizure occurrence (1 year). A resected area encompassed the right T1, amygdala and uncus, with diffuse gliosis at histopathology.

Two of the 6 new patients also underwent surgical resection. In patient no. 7, the sEEG study, following these noninvasive investigations, confirmed the presence of a right temporal pole focus (T1 and T2) which was surgically removed. The patient has had no more seizures (7 months). The surgical specimen revealed severe temporal neocortical gliosis over T1. Patient no. 10 underwent surgery (left temporal lobe) without further sEEG investigations; no seizure occurrence was reported during the postoperative follow-up (1 year). The histopathological diagnosis was hippocampal sclerosis associated with temporal neocortical gliosis. On the basis of our findings, 4 patients were deemed ineligible for surgery (patients nos. 1, 5, 8 and 9); at present writing, the remaining 2 patients are waiting to be operated on.

**Fig 6:**
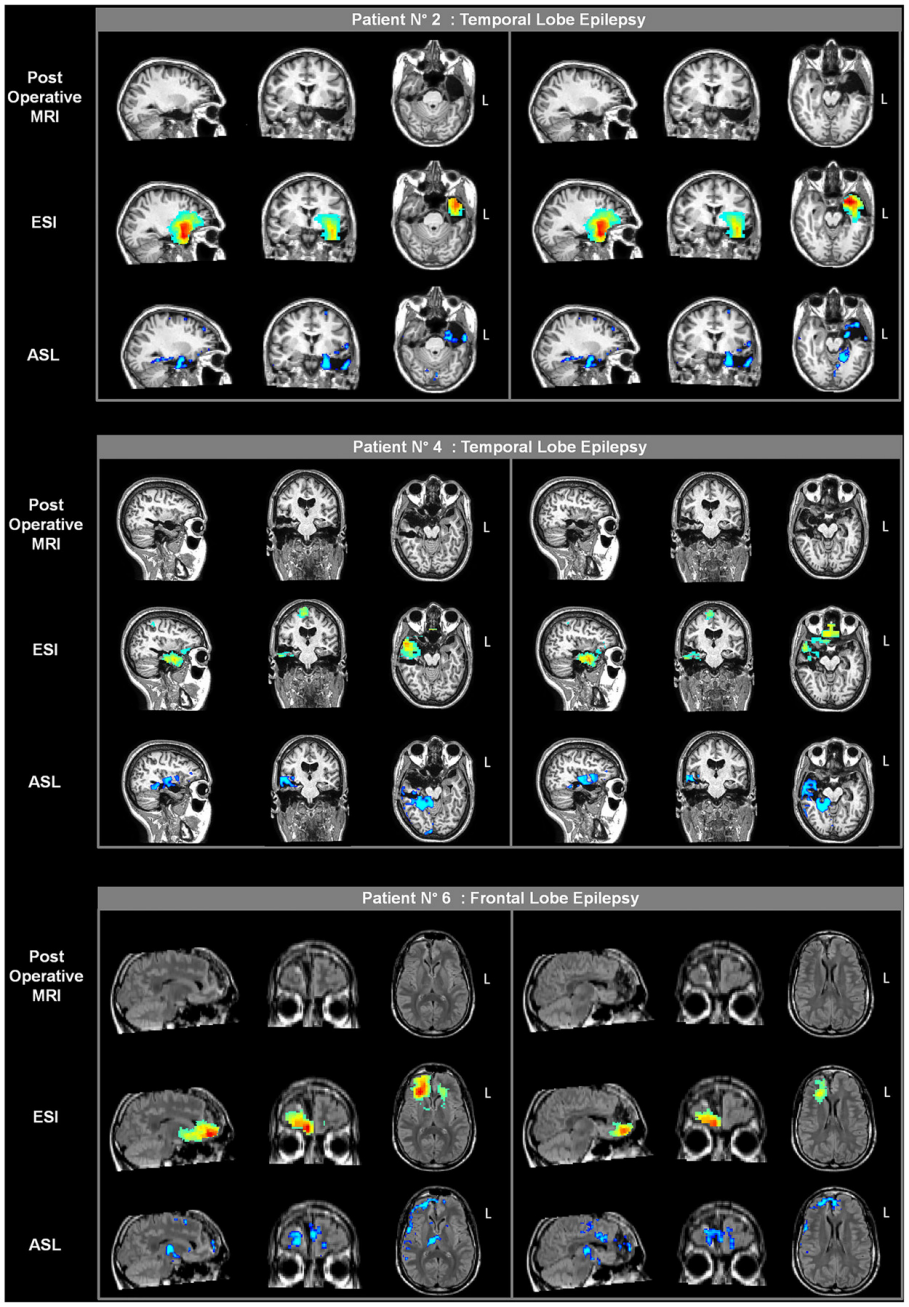
Postoperative imaging in three patients from the previously described patient group (patients nos. 2, 4 and 6). The presurgical ESI and ASL results are overlaid on the coregistered postoperative MRI scans of each patient. Two different sections are shown for each plane (sagittal, coronal and axial). The rising phase of activity and the statistical results from the one-versus-many analysis are presented for ESI and ASL, respectively.

**Fig 7:**
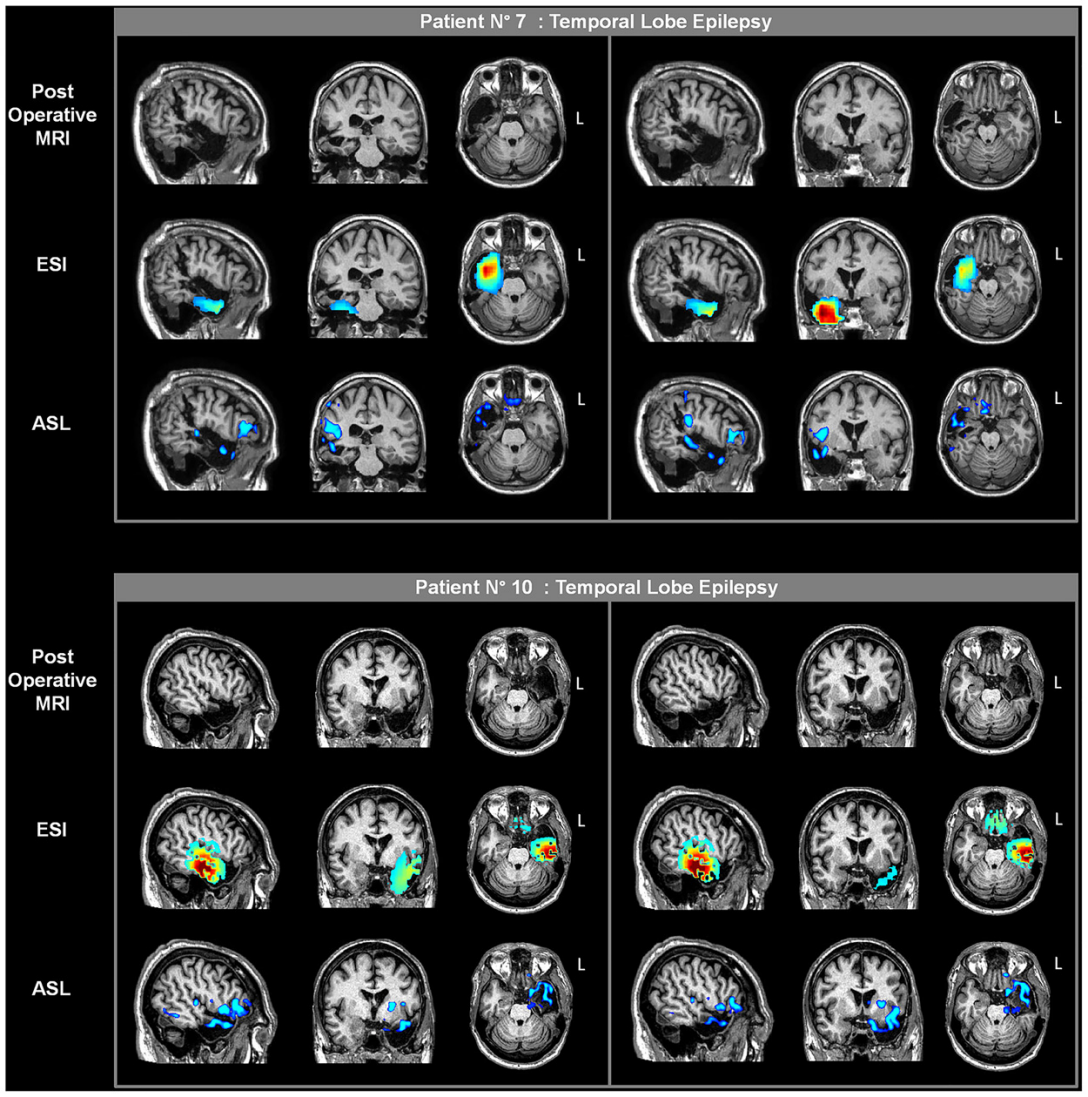
Postoperative imaging in two patients of the new group (patients. nos. 7 and 10). The presurgical ESI and ASL results are overlaid on the coregistered postoperative MRI scans for each patient. Two different sections are shown for each plane (sagittal, coronal and axial). The rising phase of activity and the statistical results from the one-versus-many analysis are presented for ESI and ASL, respectively.

The presurgical information for ESI and ASL overlaid on the postoperative MRI scan of each of the 5 patients is reported in Figs 6 and 7. With regard to the ESI results, there was good agreement between the areas of high activity, as identified by this technique, and the resected brain tissues in the 4 patients with temporal lobe epilepsy, whereas there was a non-complete concordance for the frontal lobe epilepsy patient. Indeed, ESI placed part of the highest activity more inferiorly than the resected portion of the frontal lobe, which was more superior and mesial. Conversely, in this patient ASL provided more concordant results, with hypoperfused voxels located well over the right frontomesial portion and with limited spread to other areas. A good overlap was seen between the hypoperfused regions and the resected tissues in the patients with temporal lobe epilepsy.

## Discussion

The present study reports the results of a comprehensive assessment of the feasibility of ASL in detecting perfusion changes in epilepsy. The patients’ series now includes 12 drug-resistant epileptic patients, with the addition of 6 new patients since the publication of our first report [29]. In all these patients, significant CBF changes were detected which well matched the electrophysiological information, providing further evidence that ASL can be a useful aid in identifying epileptic activity-related CBF changes. The combination of ASL perfusion MRI with an already established method as ESI estimated from the hdEEG data (256 channels) produced a clearer picture of the epileptogenic zone in terms of perfusion and current density. In this series, the ASL and ESI results were concordant and provided complementary information borne out by quantitative assessment of specific parameters (CBF and CD, respectively) in the same anatomical regions. Differently from previous papers which performed only partially quantitative analyses [38, 40–41], this is the first ASL study to quantitatively fully assess CBF via a more complete statistical approach to automatically identify perfusion alterations in individual epileptic patients, along with ROI quantification and comparison with the ESI results. In 5 of the 12 epileptic patients, the presurgical ASL and ESI results were further confirmed by the surgical excision information, with a good overlap between the preoperative information and the resected area.

ASL has seldom been employed in the evaluation of epileptic patients, even though a good accuracy in identifying the focus localization has been demonstrated by ASL in combination with other methods as EEG, PET, SPECT or gadolinium-based perfusion MRI in all of these few cases [38–39, 41–42]. Perfusion alterations in epileptic brains are a well-known phenomenon described in both human studies, mainly with SPECT techniques [30–31], and in experimental animal models [65–66], and their assessment could enormously benefit from the application of a non-invasive technique as ASL. In particular, these studies showed that the different CBF levels detected during interictal/ictal/postictal phases hint not only at variations in this parameter but also in the underlying damage to the blood-brain barrier by inflammatory processes in the epileptogenic focus.

While the previous study [29] focused mainly on comparing the results from ASL and [^18^F]FDG-PET, in the present study we further investigated the applicability of ASL in epilepsy by means of an automatic approach to reliably identify areas of epileptiform activity. Previous studies in ASL and epilepsy reported only on qualitative assessment of the CBF maps [41] or calculated the asymmetry index values between the right and the left hemispheres within ROIs manually drawn in specific portions of the brain [38–39, 42]. Here, two complementary approaches are proposed for assessing CBF maps at the patient level. We first adopted a ROI-based approach, already employed in the previous study, in order to quantitatively evaluate possible asymmetries between the two hemispheres and to obtain a preliminary picture of the main areas involved in the epileptic process. This analysis in addition allowed us to quantitatively compare the ESI and ASL results over the same anatomical regions, thus overcoming the limitations inherent to the differences in original spatial resolution and acquisition time. Nevertheless, visual analysis of the CBF maps and/or evaluation of the right-left asymmetries were difficult to interpret in some cases due to slight variations in the hemispheres or global reduction in total perfusion. For these reasons, a complementary analysis was performed, starting from the CBF maps of a control group and then constructing a normal perfusion template that served as reference for evaluating at the voxel level the individual CBF maps of each patient. This allowed us to provide the statistical significance to the previous results and more easily interpret the parametric CBF maps. In all 12 patients, the agreement between the first results from the ROI analysis and the statistical analyses was good, providing a clearer localization of the altered regions and thus of the possible epileptic focus.

This automatic approach represents a more complete tool for the one-versus-many analysis, where both within-subject and between-subject variability were taken into account in the template creation and the subsequent *t*-test. Traditionally, the importance of within-subject variance information is underestimated when a single subject is compared to a control group and variance values are not considered in the statistics [44, 67]. In particular, Petr et al. [60] presented a template-based analysis for detecting individual activation patterns in functional ASL data, which they also applied in combination with a *z*-score in an epileptic patient in order to detect hypoperfused areas associated with dysplasia. However, variance information was not taken into account in the construction of the template, where only the mean CBF maps of each subject were considered (DARTEL algorithm), and no quantitative validations were performed. A recent study by Maumet et al. [51] demonstrated, using a mixed-effect generalized hierarchical linear model, that the within-subject variance cannot be considered negligible by comparison to between-subject variance nor constant across subjects in ASL data. They in particular reported that the patient-specific brain perfusion abnormalities in a series of brain tumor patients could be correctly detected using ASL if the heterogeneous within-subject variances are properly modeled. In our study, we applied a slightly different approach, separating the construction of the normal perfusion template from the statistical analysis. In both steps, the within-subject variance was modeled in agreement with previous studies [50–51]. In the first step, the variance derived for the normal perfusion template included both within-subject and between-subject variability. Moreover, since both the mean and variance matrices for each subject were supplied to FLAME 1, the algorithm weighted the subjects according to their individual variance, so that the overall variance was a better reflection of the population. This group variance was then used, along with the patient variance, in the subsequent unpooled two-sample *t*-test to obtain a precise localization of perfusion alterations. The template-based approach allowed us to detect areas with a statistically significant decrease in perfusion during the interictal phase (10 out of 12 patients) and a statistically significant perfusion increase in the early postictal phase in the remaining 2 patients. This two-step methodology is flexible and can be easily applied to all types of ASL data, the only requirement is the creation of the own specific CBF template, since this can vary depending on the type of sequence used for the acquisition, its main parameters and the age-range of patients.

In all cases, localization of statistically significant alterations was concordant with the ESI results from the hdEEG recordings. Indeed, also in this study source imaging was used as a reliable reference to evaluate the localizing ability of ASL. To our knowledge, no previous studies, except our pilot work [29], have attempted to combine ASL-derived CBF maps with source imaging from hdEEG. In detail, ESI describes the propagation of electrical activity from an area of primary activation to other cortical regions. The electrical propagation can be estimated by analyzing the time course of the voltage fields, although only the rising phase and the peak activity were reported here by convention [24]. When the voltage fields change in amplitude but not in shape or location, this suggests a discrete source with no or limited propagation. Differently, changes in shape or amplitude reflect electrical propagation, as seen in patient no. 9 [68]. Importantly, it has to be underlined that our work was not aimed at substituting one technique (ASL) to another (ESI), but to support results soundness by a bidirectional concordance in drug-resistant epileptic patients who are candidate for surgery. In addition, due to the different signal source generators (electrical signal vs CBF) no perfect overlap between ESI and ASL results can be expected, pinpointing a complementarity rather than the interchangeability of the two methods. In particular, ESI is a modeled reconstruction of the cortical area generating the spike signal in a specific instant or time window, while ASL like PET encompasses a broader time frame and provides an average measure of any event that has occurred during acquisition. A multimodal approach can thus allow to obtain more reliable and complementary information to guide the identification of the epileptogenic zone, and not redundant ones, while compensating for the imprecision inherent to each modality.

Whereas the robustness of ESI localization in epilepsy has been widely studied during the years and has been supported by the combination of invasive EEG (stereo-EEG) and postoperative results [17,19,64,69], depicting ESI as a reliable tool to identify the seizure onset zone to be resected, ASL validation and application for this pathology is still matter of research. However, the few available literature show promising results and, despite the limited number of patients, our results could help to further confirm ASL usefulness in epilepsy, especially in critical cases where ESI can have low sensitivity. Indeed, previously published data report on a limited sensitivity of ESI in detecting sources located in deeper gray matter areas—typically mesial temporal spikes, but also sources embedded in intricate circumvolutions and far from the skull, as can be for orbito-mesio-frontal regions. It seems that the major reasons for the limited sensitivity of the EEG to these spikes are the rapid decay of electrical activities with distance and the convoluted anatomy of deep lobe structures that tends to create electric/magnetic fields in close proximity [17]. Therefore, especially in these cases where ESI shows lower sensitivity and in patients with complex epilepsy patterns, additional non-invasive and quantitative information can be derived from ASL for a more precise focus localization.

The present investigation allowed us to correctly identify the epileptogenic zone in 5 patients (patients nos. 2, 4, 6, 7, and 10), in which the results were confirmed by the surgical resection and seizure freedom (gold standard). In another patient (patient no. 3), the ASL and ESI results were also confirmed by seizure freedom following radiofrequency thermocoagulation during a sEEG investigation performed on the basis of our findings. Information gleaned from postsurgical MRI scans and seizure freedom are essential for determining the localization accuracy of ASL and ESI techniques. In the temporal lobe epilepsy patients, for example, there was a good overlap between the resected areas and the presurgical ESI and ASL results in all cases, demonstrating that both techniques concur in correct focus localization. In some cases, as in patient no. 7, the area of altered perfusion appeared to be much smaller on the ASL image than the actual extent of the lesion, though this apparent discordance might also be attributed to the restrictive FDR correction applied to the statistical results. In the frontal lobe epilepsy patient, a non-complete overlap was detected. Indeed, part of the ESI was located more inferiorly than the resected portion of the frontal lobe, which was more superior and mesial, and contrary to ASL images which provided more concordant results. As reported elsewhere [17, 69–70], such cases pose diagnostic dilemmas probably because of the geometrical and cytoarchitectonic characteristics of the generator. Variations in the seizure generator may explain why it is so difficult to accurately localize the focus in mesial frontal epilepsy and confirm why a multimodal approach to presurgical localization of the focus in different types of epilepsy is important.

Collectively, our results suggest that, differently from source imaging, perfusion alterations generally involve a series of areas. This is in agreement with previous studies with [^18^F]FDG-PET which generally showed areas of hypometabolism in regions beyond the temporal and frontal lobe in patients with temporal and frontal lobe epilepsy, respectively [14, 71–72]. An intriguing hypothesis could be that areas of altered perfusion extending beyond the limits of the epileptogenic zone could reflect subtler neuronal alterations, and thus delineate the so-called irritative zone, which, as defined by Luders et al. [73], is the area of cortex that generates interictal spikes, as opposed to the epileptogenic zone defined as the “minimal area of cortex that must be resected to produce seizure freedom”. Moreover, ASL as well as PET images of regional perfusion and metabolism can be interpreted as evidence for a dysfunctional epileptic network. This concept emerged in the last decade [74], and from it stems the definition of “system epilepsy”, highlighting that it is not the isolated epileptic focus that generates the full blown seizure, but rather the additive result of subsequent activation of multiple cortical areas that determines seizure semeiology.

In conclusion, multiple imaging modalities in the same patient allow for a more accurate and precise identification of the epileptogenic zone, providing better surgical outcomes and reduced postoperative deficits. The use of completely noninvasive techniques such as ESI and ASL could reduce the need for more expensive and risky sEEG investigations in candidates for surgery or allow for better targeting of intracranial electrode implantations if deemed necessary. The combined use of these two techniques shows good sensitivity in localizing the epileptic focus in relation to the resected zone identified on the postoperative anatomical scan and provides complementary electrical and perfusion information to better understand the underlying pathological mechanisms.
